# Bacteria Modify *Candida albicans* Hypha Formation, Microcolony Properties, and Survival within Macrophages

**DOI:** 10.1128/mSphere.00689-20

**Published:** 2020-08-05

**Authors:** Ornella Salvatori, Rohitashw Kumar, Sarah Metcalfe, Margaret Vickerman, Jason G. Kay, Mira Edgerton

**Affiliations:** a Department of Oral Biology, School of Dental Medicine, University at Buffalo, Buffalo, New York, USA; University of Georgia

**Keywords:** *Candida albicans*, *Pseudomonas aeruginosa*, *Streptococcus gordonii*, hyphal development, macrophages, microcolonies

## Abstract

Phagocytic cells are crucial components of the innate immune system preventing Candida albicans mucosal infections. Streptococcus gordonii and Pseudomonas aeruginosa often colonize mucosal sites, along with C. albicans, and yet interkingdom interactions that might alter the survival and escape of fungi from macrophages are not understood. Murine macrophages were coinfected with S. gordonii or *P. aeruginosa*, along with C. albicans to evaluate changes in fungal survival. S. gordonii increased C. albicans survival and filamentation within macrophage phagosomes, while *P. aeruginosa* reduced fungal survival and filamentation. Coinfection with S. gordonii resulted in greater escape of C. albicans from macrophages and increased size of fungal microcolonies formed on macrophage monolayers, while coinfection with *P. aeruginosa* reduced macrophage escape and produced smaller microcolonies. Microcolonies formed in the presence of *P. aeruginosa* cells outside macrophages also had significantly reduced size that was not found with *P. aeruginosa* phenazine deletion mutants. S. gordonii cells, as well as S. gordonii heat-fixed culture supernatants, increased C. albicans microcolony biomass but also resulted in microcolony detachment. A heat-resistant, trypsin-sensitive pheromone processed by *S. gordonii* Eep was needed for these effects. The majority of fungal microcolonies formed on human epithelial monolayers with *S. gordonii* supernatants developed as large floating structures with no detectable invasion of epithelium, along with reduced gene expression of C. albicans *HYR1*, *EAP1*, and *HWP2* adhesins. However, a subset of C. albicans microcolonies was smaller and had greater epithelial invasiveness compared to microcolonies grown without *S. gordonii*. Thus, bacteria can alter the killing and escape of C. albicans from macrophages and contribute to changes in C. albicans pathogenicity.

**IMPORTANCE** Candida albicans is the predominant fungus colonizing the oral cavity that can have both synergistic and antagonistic interactions with other bacteria. Interkingdom polymicrobial associations modify fungal pathogenicity and are believed to increase microbial resistance to innate immunity. However, it is not known how these interactions alter fungal survival during phagocytic killing. We demonstrated that secreted molecules of S. gordonii and P. aeruginosa alter C. albicans survival within the phagosome of macrophages and alter fungal pathogenic phenotypes, including filamentation and microcolony formation. Moreover, we provide evidence for a dual interaction between S. gordonii and C. albicans such that S. gordonii signaling peptides can promote C. albicans commensalism by decreasing microcolony attachment while increasing invasion in epithelial cells. Our results identify bacterial diffusible factors as an attractive target to modify virulence of C. albicans in polymicrobial infections.

## Abstract (precis)

Candida albicans is the predominant fungus colonizing the oral cavity that can have both synergistic and antagonistic interactions with other bacteria. Interkingdom polymicrobial associations modify fungal pathogenicity and are believed to increase microbial resistance to innate immunity. However, it is not known how these interactions alter fungal survival during phagocytic killing. We demonstrated that secreted molecules of S. gordonii and P. aeruginosa alter C. albicans survival within the phagosome of macrophages and alter fungal pathogenic phenotypes, including filamentation and microcolony formation. Moreover, we provide evidence for a dual interaction between S. gordonii and C. albicans such that S. gordonii signaling peptides can promote C. albicans commensalism by decreasing microcolony attachment while increasing invasion in epithelial cells. Our results identify bacterial diffusible factors as an attractive target to modify virulence of C. albicans in polymicrobial infections.

## INTRODUCTION

Oral candidiasis is a superficial mucosal infection by Candida albicans that appears macroscopically as white lesions and microscopically as interconnected radiating hyphae originating from single cells termed microcolonies (1). Microcolonies are a more virulent form of fungal growth due to their extensive hyphae that invade epithelial cells, as well as their high expression of several virulence genes, including *ECE1* (encoding candidalysin, a peptide toxin critical for mucosal infection), *HYR1* (encoding a hyphal wall protein that modulates phagocytic killing activity), and *HWP1* (encoding a hyphal wall protein that mediates tight binding to oral epithelial cells) (2).

In the oral environment, C. albicans typically resides in complex polymicrobial communities, along with multiple bacterial and other fungal species. Interactions between C. albicans and single bacterial species may be synergistic or antagonistic and have been well described in excellent reviews (3–5). C. albicans exhibits cooperative relationships with multiple oral streptococci but has a particularly high affinity for Streptococcus gordonii (6, 7), a Gram-positive bacterium that is one of the first colonizers of the oral cavity (8). Coinfection of C. albicans and oral streptococci in a murine model of oral candidiasis increases the severity of fungal lesions (9), and C. albicans biofilms formed *in vitro* were synergistically increased by streptococci (7, 10). In addition to physical interactions, both species respond to signaling metabolites, including quorum-sensing molecules. For example, autoinducer 2 secreted by S. gordonii relieves farnesol-induced suppression of C. albicans hyphal formation within cospecies biofilms (7), so that C. albicans virulence and invasion into epithelial tissues is promoted. The role of S. gordonii in C. albicans hyphal morphogenesis and virulence is further supported by a transcriptional study by Dutton et al. showing that coculture of C. albicans with S. gordonii increases expression of genes required for morphogenesis (*TEC1* and *ALS1*) and oxidative stress response (*CAT1*) (11).

In contrast with S. gordonii, interactions between Pseudomonas aeruginosa and C. albicans are mainly antagonistic (12, 13). P. aeruginosa is commonly isolated as part of mixed infections with C. albicans from the lungs of cystic fibrosis patients (14) and as a part of the oral microbiota (15). P. aeruginosa, a Gram-negative opportunistic rod, is becoming increasingly important in clinical settings because of its antibiotic resistance (16). Clinical isolates of P. aeruginosa are able to reduce C. albicans survival (17) and kill germinated C. albicans cells (18, 19) due to secretion of bacterial phenazines (20). Besides its candidacidal activity, P. aeruginosa influences C. albicans morphology by reducing filamentation (21), and can adhere to C. albicans hyphal cells in response to a secreted quorum-sensing molecule, 3-oxododecanoyl-l-homoserine lactone (22). This antagonistic interaction is bilateral, since C. albicans can produce farnesol that reduces P. aeruginosa secretion of pyocyanin (23) and motility (24). Importantly, prior colonization of lungs by C. albicans increased the clearance of subsequent P. aeruginosa infection via leukocyte recruitment (25).

Key components of innate immunity are phagocytic cells, including macrophages that recognize and engulf C. albicans and other microbes into phagosomes where they are killed (26–28). C. albicans, as well as bacteria, can survive phagocytic killing (29–31) by suppressing phagosomal maturation (31) or reactive oxygen species (ROS) production (32, 33). Despite the well-known relationships of C. albicans with S. gordonii and P. aeruginosa, it is unclear whether simultaneous phagocyte interactions with yeast and bacteria affects the subsequent survival of C. albicans. Phagocytosis of S. gordonii alone by activated macrophages can permit phagosomal disruption on maturation, resulting in increased bacterial survival (34), and P. aeruginosa can evade phagocytosis by modifying its motility (35, 36). It is likely that cospecies interactions within phagocytes will further modify their survival.

In this study, we investigated the effect of these two bacteria on C. albicans after phagocytosis by macrophages. We show for the first time that phagocytosis of C. albicans in the presence of selected strains of these bacteria altered the survival of C. albicans within macrophages that was associated with induction or repression of fungal hyphae. P. aeruginosa decreased fungal survival in macrophages and inhibited C. albicans filamentation and microcolony formation, while S. gordonii increased survival and C. albicans hyphal formation and their escape from macrophage. C. albicans microcolony density and biomass were also significantly altered by products of both bacteria. Phenazines produced by P. aeruginosa reduced microcolony biomass, while S. gordonii culture supernatants increased biomass, but simultaneously caused loss of adhesion of fungal microcolonies to the underlying substrate and reduced epithelial invasion. Coincubation with S. gordonii also led to altered expression of some C. albicans microcolony virulence genes, suggesting that S. gordonii can modify microcolony virulence.

## RESULTS

### Bacterial species modify *C. albicans* survival within murine macrophages.

**FIG 1:**
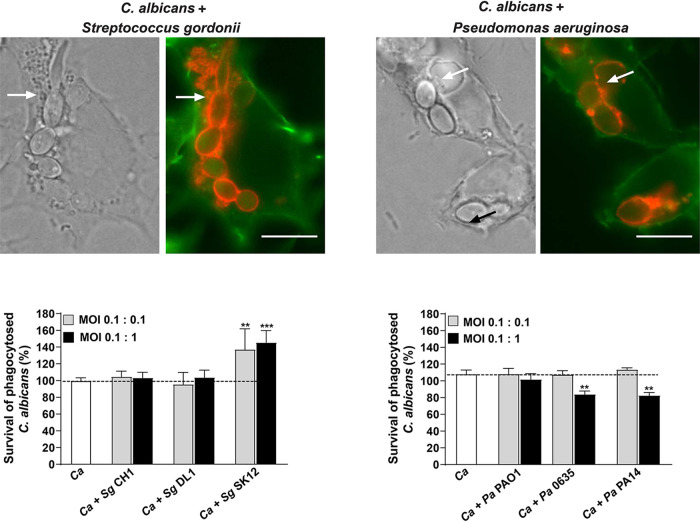
S. gordonii SK12 strain increases C. albicans survival in macrophages, while P. aeruginosa 0635 and PA14 decrease C. albicans survival. **(**Upper panels) Phagocytosed C. albicans and bacterial species by murine RAW 264.7 macrophages were evaluated microscopically after 30 min of incubation. Macrophages were stained with phalloidin (green) and phagosomes were immunostained for LAMP1 (red). White arrows indicate phagocytosed S. gordonii SK12 (left) and P. aeruginosa 0635 (right). Scale bar, 10 μm. (Bottom panels) Macrophages were infected with *C. albicans* and S. gordonii CH1, DL1, and SK12 strains (left) or *P. aeruginosa* PAO1, 0635, and PA14 strains (right) at an MOI of 0.1:0.1 (gray bars, 0.1 *C. albicans*:0.1 bacteria) or an MOI of 0.1:1 (black bars, 0.1 *C. albicans*:1 bacteria). After 3 h of coincubation, macrophages were lysed, and internalized *C. albicans* was released and plated on antibiotic supplemented agar to remove all bacteria and obtain *C. albicans* CFU. Survival was calculated as follows: (recovered *C. albicans* CFU from macrophages/total number of phagocytosed *C. albicans*) × 100. Coincubation with S. gordonii SK12 significantly increased *C. albicans* survival, while *P. aeruginosa* 0635 and PA14 significantly decreased *C. albicans* survival. Means ± the standard deviations (SD) of at least three independent experiments carried out in duplicate are shown. Significance was obtained using one-way ANOVA with *post ad hoc* Dunnett’s multiple-comparison test (**, *P* < 0.01; ***, *P* < 0.001). Labels: *Ca*, *C, albicans*; *Sg*, *S. gordonii*; *Pa*, *P. aeruginosa*.

S. gordonii (strains CH1, SK12, and DL1), a Gram-positive coccus, and P. aeruginosa (strains PAO1, 0635, and PA14), a Gram-negative rod, were tested for their ability to modify C. albicans survival and escape from macrophages. Either *S. gordonii* or *P. aeruginosa* species were added with C. albicans so that 95 to 100% of *C. albicans* and 90 to 95% of bacteria were phagocytosed (multiplicities of infection [MOIs] of 0.1 *C. albicans*:0.1 bacteria or 0.1 *C. albicans*:1 bacteria), as shown microscopically (Fig. 1, upper panels). The phagocytic index of *C. albicans* in macrophages at these ratios was not altered by the presence of either bacterial species (see Fig. S1 in the supplemental material). *C. albicans* and bacteria appeared to be colocalized together within phagosomes of macrophages when visualized microscopically (Fig. 1, upper panels, white arrows), although we could not ascertain their absolute proximity. At 3 h after coinfection, neither *S. gordonii* CH1 or *S. gordonii* DL1 had significantly altered *C. albicans* survival within phagosomes compared to *C. albicans* alone (*C. albicans* survival of 99.7%; Fig. 1, lower left panel). In contrast, coinfection with *S. gordonii* SK12 significantly increased survival (137 to 145%) of *C. albicans* after 3 h compared to the control. In contrast, cospecies infection of *C. albicans* with *P. aeruginosa* clinical isolates (0635 or PA14) resulted in a 23% decrease in *C. albicans* survival after 3 h compared to *C. albicans* alone, which was not observed with the laboratory strain of *P. aeruginosa* (PAO1).

10.1128/mSphere.00689-20.1 FIG S1 C. albicans and bacterial coinfection did not alter the phagocytic index in macrophages. For phagocytosis assays, adherent phagocytic cells (RAW 264.7) were seeded in 12-well plates with cover glasses (Azer Scientific) for 15 min to allow cells to attach. C. albicans and either S. gordonii or P. aeruginosa whose growth was synchronized to mid-log phase were suspended in RMPI 1640 and added to phagocytic cells at an MOI of 0.1:01 or 0.1:1 (yeast and bacteria) and then incubated for 30 min at 37°C with 5% CO_2_ to allow phagocytosis. After incubation, cover glasses were washed with ice-cold PBS to remove medium, and 4 mg/ml of calcofluor white in PBS (CW; Sigma-Aldrich) was added for 2 min on ice to stain nonphagocytosed C. albicans. Next, cover glasses were washed with ice-cold PBS to remove excess of CW and fixed with 4% paraformaldehyde (Electron Microscopy Sciences) for 30 min at room temperature. After fixation, the cells were permeabilized with 0.1% Triton X-100 (Fisher Bioreagents) for 5 min and stained with 4 mg/ml Alexa Fluor 488-conjugated phalloidin (Invitrogen) for 5 min. After a final wash, cover glasses were mounted to slides (Globe Scientific, Inc.), and positively charged slides were covered with a number 1 cover glass (Knittel Glaser) using fluorescent mounting media (Dako). Cells were counted using a Zeiss Axio Observer Z1 inverted fluorescent microscope (Carl Zeiss, Germany). A minimum of 100 phagocytic cells were observed for each experiment, and internalized C. albicans cells that were not stained with CW were counted. The percent phagocytosis was calculated as the ratio of total number of phagocytosed C. albicans, and the total number of phagocytes was counted. Assays were performed in duplicates, and experiments were repeated at least three times. Labels: *Ca*, *C, albicans*; *Sg*, *S. gordonii*; *Pa*, *P. aeruginosa*. Download FIG S1, EPS file, 2.7 MB. Copyright © 2020 Salvatori et al. 2020 Salvatori et al. This content is distributed under the terms of the Creative Commons Attribution 4.0 International license.

### *S. gordonii* promoted and *P. aeruginosa* repressed *C. albicans* hyphal formation within macrophages.

**FIG 2:**
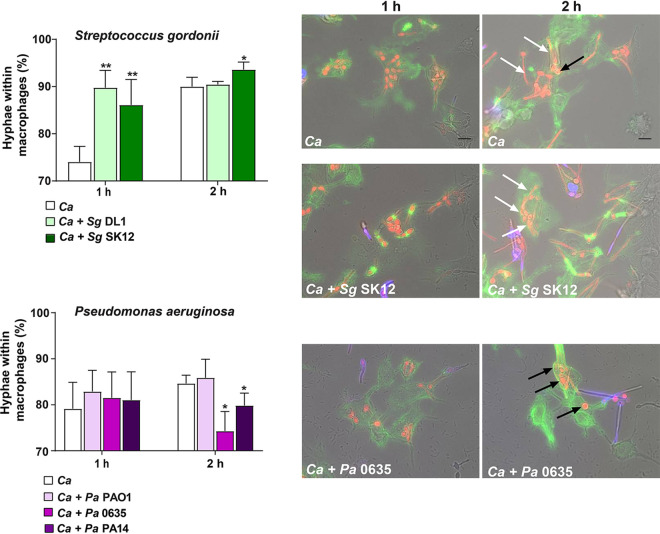
C. albicans hypha formation within macrophages is altered in the presence of bacteria. C. albicans CAF2-dTomato was added in an MOI of 0.5 to RAW 264.7 macrophages in the presence of S. gordonii (upper panels) or P. aeruginosa strains (lower panels). Macrophages and C. albicans were incubated without bacteria as a control. Nonphagocytosed C. albicans were stained with calcofluor white (blue), and macrophages were stained with phalloidin (green). At least 100 phagocytosed C. albicans (red) were counted and classified morphologically as yeast or hyphae microscopically. The percentage of hyphal cells was calculated by obtaining the ratio of total C. albicans with hyphae/total number of C. albicans counted × 100. C. albicans long hyphae (white arrows) or short hyphae (black arrows) within macrophages are shown with or without bacteria (right panel). Experiments were carried out in duplicate, and graphs represent the means ± the SD of three independent experiments. Significance was obtained by one-way ANOVA with *post ad hoc* Dunnett’s multiple-comparison test (*, *P* < 0.05; **, *P* < 0.01). Scale bar, 10 μm.

Since C. albicans is known to initiate hyphal formation in macrophages in order to escape killing (37), we next examined whether the presence of bacteria altered C. albicans hypha formation within the lumens of phagosomes. To compare hyphal length outside macrophages to that inside phagosomes, we used a higher MOI (0.5 *C. albicans* alone or 0.5 *C. albicans*:5 bacteria) so that some C. albicans (and bacteria in coinfection experiments) would not be phagocytosed, allowing us to compare germination outside macrophages with that within phagosomes. Surprisingly, the presence of *S. gordonii* DL1 (*Sg*DL1) or *Sg*SK12 both resulted in a significant (*P* < 0.01) increase in phagosomal *C. albicans* hyphal formation (89 to 86% hyphal formation with bacteria compared to 75% germination with *C. albicans* monoinfection) after 1 h (Fig. 2, left upper panel). After 2 h, there was no statistical difference between number of germinated cells between monoinfection and coinfection. However, 2 h after coinfection with *S. gordonii*, most *C. albicans* within the phagosome formed very elongated hyphae (white arrows) compared to monoinfection in which many *C. albicans* had very short hyphae (black arrows, Fig. 2, upper panel). In comparison, extracellular nonphagocytosed *C. albicans* (Fig. 2, *Ca+Sg* panel, purple stain) formed similar long hyphae as phagosome-localized *C. albicans* in the presence of *S. gordonii*. Also, at 2 h after coinfection with *S. gordonii*, many intracellular *C. albicans* hyphae could be seen penetrating the phagosome so that the distal tips of hyphae were outside the macrophage. In monoinfections with *C. albicans* alone, most hyphae did not have sufficient length after 2 h to penetrate macrophages.

In contrast, the presence of *P. aeruginosa* had no effect on phagosomal *C. albicans* germination at 1 h, but after 2 h *P. aeruginosa* 0635 and PA14 significantly (*P* < 0.05) reduced hypha formation compared to monoinfection of *C. albicans* (Fig. 2, lower panel). Microscopically, coinfection with *P. aeruginosa* reduced hyphal length of *C. albicans* within the phagosome compared to extracellular *C. albicans*; and few hyphae were seen to escape macrophages at 2 h. To better quantify these observations, we next tested the ability of *C. albicans* to escape from macrophages and form microcolonies after coinfection with either *S. gordonii* or *P. aeruginosa*.

### Bacteria modify escape of *C. albicans* from macrophages and alter the size of resulting *C. albicans* microcolonies.

**FIG 3:**
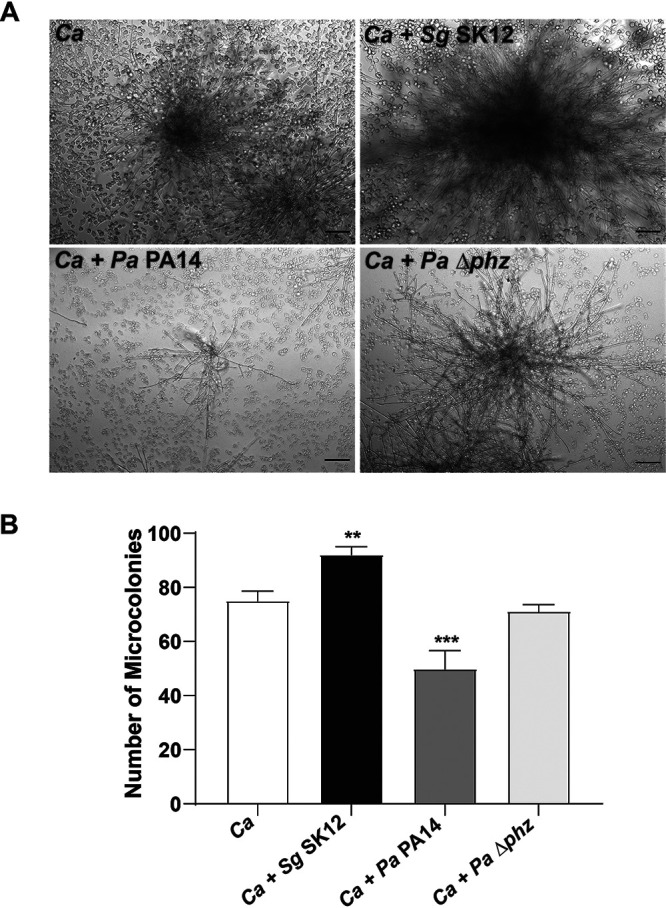
Bacteria alters microcolonies formed by phagocytosed and escaped C. albicans. (A) A total of 100 C. albicans cells were added to activated murine RAW 264.7 macrophages monolayers in the presence of 1,000 cells of S. gordonii (upper right) or P. aeruginosa strains (wild type and phenazine mutant [*Δphz*], lower panels) for 17 h. The cells were allowed to phagocytose for 3 h. Bacterial growth was controlled by adding 1× antibiotic solution. After incubation for 24 h, the microcolonies were imaged using bright-field microscopy. (B) The total number of microcolonies was counted for each well. Each experiment was carried out in duplicate, and graphs represent the means ± the SD of three independent experiments. Significance was obtained by one-way ANOVA with *post ad hoc* Dunnett’s multiple-comparison test (**, *P* < 0.01; ***, *P* < 0.001). Scale bar, 100 μm.

In order to measure relative escape ratios, macrophages were infected at an MOI of 1 macrophage: 0.1 *C. albicans*: 0.1 bacteria (or 1 macrophage: 0.1 *C. albicans* for monoinfection) to ensure nearly complete phagocytosis of both yeast and bacteria, incubated together for 3 h, and then streptomycin and penicillin were added to suppress the growth of escaped or nonphagocytosed bacteria. Macrophage monolayers and fungal cells then were grown at 37°C in 5% CO_2_ for 17 h to permit visualization of C. albicans able to form microcolonies following escape from macrophages. C. albicans cells from monoinfections formed clearly visible microcolonies that grew on top of macrophages without damaging the majority of underlying cells (Fig. 3A), suggesting that the growth of escaped fungal cells did not result in lysis of nearby macrophages. The mean escape ratio for *C. albicans* monoinfection was 76 microcolonies per 100 added *C. albicans* (Fig. 3B)*. C. albicans* and *Sg*DL1 coinfection resulted in the formation of microcolonies that were much larger and denser than for those of monoinfection alone and increased the escape ratio to 96/100. *C. albicans* and *P. aeruginosa* PA14 (*Pa*PA14) coinfection led to a much lower escape ratio of 55/100, and the microcolonies formed were much smaller and less dense. Since phenazine production by *P. aeruginosa* is known to inhibit filamentation, we examined a *P. aeruginosa* phenazine mutant (*Δphz*) expecting that these cells would more closely resemble monoinfection by *C. albicans*. Indeed, *Δphz* cells had a significantly higher escape ratio (70/100) and formed denser and larger microcolonies than did the parental *P. aeruginosa* strain, suggesting that phenazine production by *P. aeruginosa* is largely responsible for the reduction in *C. albicans* microcolony size and reduced escape from macrophages.

### Maturation of *C. albicans*-containing phagosomes and ROS production was not altered by *S. gordonii* and *P. aeruginosa*.

To test whether changes in killing and escape of *C. albicans* from macrophages after coinfection with bacteria may be a result of changes in phagosomal maturation or ROS production, we measured the maturation kinetics of *C. albicans*-containing phagosomes by the loss of EEA1 (a marker of early maturation) and the acquisition of LAMP1 or lysosome-chased dextran (markers of phagosomal maturation) (34, 38). Coinfection of macrophages with *C. albicans* and either *S. gordonii* or *P. aeruginosa* did not change the rate of phagosomal maturation, as measured by the loss of EEA1 or the increase of dextran. Acquisition of LAMP1 differed only for one strain of *S. gordonii* (SK12) (see Fig. S2), in total showing that *C. albicans* survival and escape from macrophages in the presence of bacteria was not likely to be altered by phagosomal maturation. Since production of ROS by phagosomes is an important killing mechanism (39), we also examined the total release of ROS over 2.5 h from macrophages incubated with *C. albicans* in combination with *S. gordonii* or *P. aeruginosa*. There were no significant differences in ROS in macrophages coinfected with *C. albicans* and either *S. gordonii* or *P. aeruginosa* strains compared to monoinfection with *C. albicans* alone (Fig. S2). Thus, altered C. albicans survival in macrophages in the presence of bacteria did not appear to be a result of altered ROS production or delayed phagosomal maturation within the initial 90 to 180 min after coinfection.

10.1128/mSphere.00689-20.2 FIG S2 S. gordonii SK12 decreased maturation of phagosomes containing C. albicans. (A) Phagosomes of RAW 264.7 containing C. albicans with S. gordonii (left upper panel) or P. aeruginosa strains (left lower panel) were stained with LAMP1 antibody, and positive phagosomes were quantified microscopically. The percentage of LAMP1-positive phagosomes was calculated as follows: (number of positive phagosomes/total number of phagosomes counted) × 100. Macrophages and C. albicans were incubated without bacteria as control. Macrophages with LAMP1-positive (red arrows) and -negative (white arrows) phagosomes containing C. albicans incubated with or without bacteria after 30 min of coincubation are shown (right panel). Experiments were carried out in duplicate, and the means ± the SD of two independent experiments are shown. The significance between *C. albicans* and *C. albicans* + S. gordonii SK12 was obtained by one-phase association fitting (***, *P* < 0.001). Scale bar, 10 μm. ROS production by macrophages upon activation with C. albicans and bacterial strains was detected with chemiluminescence using luminol at 37°C over 2.5 h. (B) Total ROS production was calculated by obtaining the area under the curve of four independent experiments performed in duplicate, and graphs represent the means ± the SD of four independent experiments. As a control, macrophages were incubated with C. albicans without bacteria or with PMA (positive control). Download FIG S2, EPS file, 2.8 MB. Copyright © 2020 Salvatori et al. 2020 Salvatori et al. This content is distributed under the terms of the Creative Commons Attribution 4.0 International license.

### *C. albicans* microcolonies are modified by secreted molecules of *P. aeruginosa* and *S. gordonii*.

**FIG 4:**
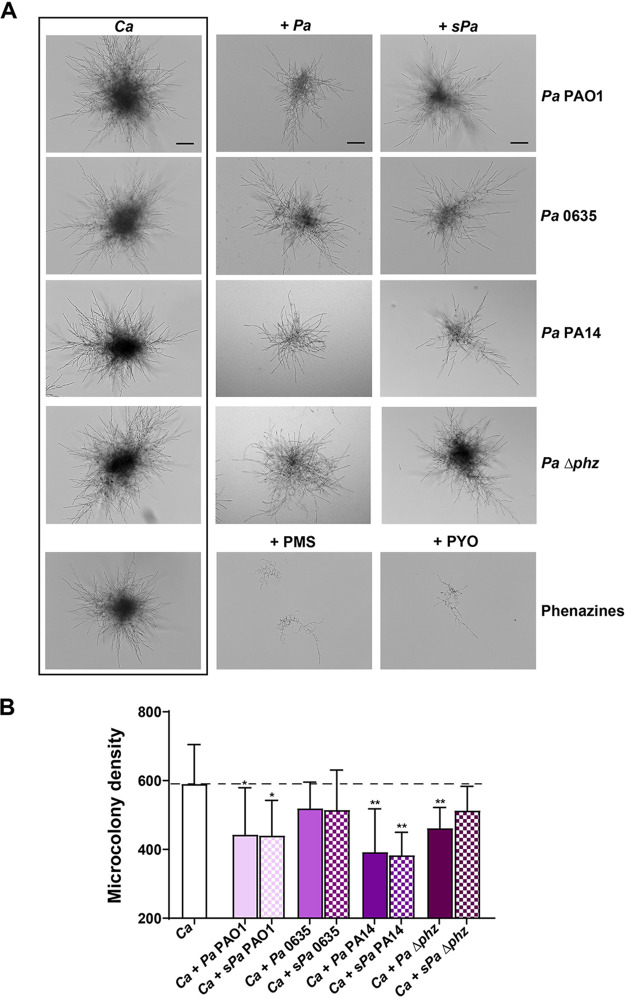
C. albicans microcolony density is decreased by P. aeruginosa and phenazines. (A) C. albicans microcolonies were cultured using RPMI 1640 medium at 37°C in 5% CO_2_, along with P. aeruginosa strains POA1, 0635, or PA14 or of fresh-filtered P. aeruginosa culture supernatant (10%; s*Pa*), phenazine methosulfate (PMS; 5 μM), or pyocyanin (PYO; 20 μM) for 17 h. C. albicans incubated alone was used as control. Images were obtained using bright-field microscopy, and the microcolony density was calculated using ImageJ. (B) The microcolony density per square micron was obtained by using ImageJ. The addition of three *P. aeruginosa* strains or s*Pa* each significantly decreased microcolony density compared to C. albicans grown in culture media alone. Experiments were carried out in duplicate, and the means ± the SD of four independent experiments are shown. Significance was obtained by Student *t* test or one-way ANOVA with *post ad hoc* Dunnett’s multiple-comparison test (*, *P* < 0.05; **, *P* < 0.01; ****, *P* < 0.0001). Scale bar, 100 μm.

Since these data suggested a role for secreted bacterial products in altering *C. albicans* filamentation within macrophages, we tested whether either bacteria themselves or secreted products modified microcolony formation outside macrophages. For these experiments, *C. albicans* microcolonies were grown on solid surfaces in the presence of bacteria or bacterial supernatants to allow quantification of growth. Similar to what we observed within macrophages, *C. albicans* microcolonies formed in the presence of three *P. aeruginosa* strains (PAO1, Pa0635, and PA14) were smaller and had fewer hyphal projections (Fig. 4A), and addition of cell-free *P. aeruginosa* culture supernatants (s*Pa*) resulted in nearly 50% reduction in microcolony density for PAO1 or PA14 strains (Fig. 4B). Quorum-sensing molecules secreted by *P. aeruginosa* play an important role during polymicrobial interactions (22, 24) and, among them, phenazines are known to suppress C. albicans hyphal morphogenesis (19, 20). We therefore decided to further examine the role of phenazines by using a *P. aeruginosa* phenazine knockout (*Δphz*) mutant, along with synthetic the methylphenazinium analogs phenazine methosulfate (PMS) and pyocyanin (PYO) that suppress hypha morphogenesis in agar (40). The addition of *Δphz* cells still resulted in some reduction in *C. albicans* microcolony size, but the *P. aeruginosa Δphz* culture supernatants did not inhibit microcolony formation consistent with the loss of secreted phenazines (Fig. 4). The addition of purified phenazines PMS (5 μM) or PYO (20 μM) almost completely blocked microcolony formation (Fig. 4A), showing that phenazines suppress *C. albicans* filamentation and repress microcolony formation.

**FIG 5:**
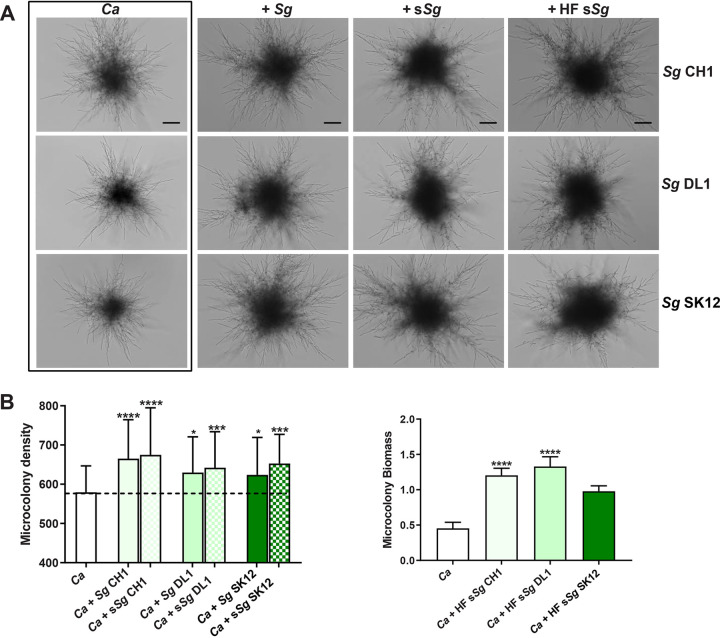
C. albicans microcolony size and density are increased by the addition of S. gordonii cells or S. gordonii supernatants. (A) C. albicans microcolonies were cultured using RPMI 1640 medium at 37°C in 5% CO_2_, along with S. gordonii strains CH1, DL1, and SK12; fresh-filtered S. gordonii culture supernatant (10%; s*Sg*); or heat-fixed (HF s*Sg*) S. gordonii culture supernatant (10%) for 17 h. Images were obtained using bright-field microscopy, and the microcolony density was calculated using ImageJ. The addition of S. gordonii or s*Sg* each significantly increased microcolony density compared to C. albicans grown in culture media alone (B, right panel). Microcolony biomass (as determined with crystal violet staining) when grown with HF s*Sg* were significantly increased with S. gordonii strains CH1, DL1, and SK12 (B, right panel). Experiments were carried out in duplicate, and the means ± the SD of four independent experiments are shown. Significance was obtained by one-way ANOVA with *post ad hoc* Dunnett’s multiple-comparison test (*, *P* < 0.05; ***, *P* < 0.001; ****, *P* < 0.0001). Scale bar, 100 μm.

Since we observed promotion of germination by *S. gordonii* cells in macrophages, we expected a similar effect on microcolony formation. Indeed, the addition of *S. gordonii* CH1, DL1, and SK12 cells, cell supernatants, and heat-fixed (HF) supernatants all increased the size of *C. albicans* microcolonies by increasing hyphal length and number of radiating hyphae (Fig. 5A). Quantification of microcolony density showed a significant increase in density between 25 and 50% among *S. gordonii* strains and with only fresh culture supernatants compared to *C. albicans* alone (Fig. 5B, left). However, we also noticed that microcolonies formed in the presence of *S. gordonii* lost their ability to adhere to the substrate, a phenomenon we did not find with *P. aeruginosa* experiments. Incubation with HF supernatants from each of the three *S. gordonii* strains with *C. albicans* that resulted in microcolonies that were so loosely adherent that they could not be quantitated by ImageJ. Instead, the total biomass of microcolonies was measured by crystal violet (CV) staining. The HF supernatants of all *S. gordonii* strains significantly increased *C. albicans* microcolony biomass by 3-fold compared to untreated *C. albicans* cells (Fig. 5B, right panel).

### *S. gordonii* Eep contributes to *C. albicans* hypha formation and microcolony biomass.

**FIG 6:**
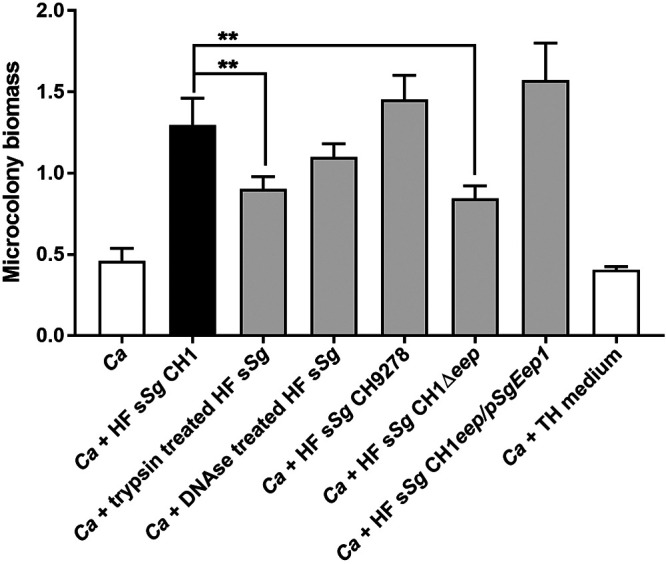
C. albicans microcolony biomass is increased by a small peptide processed by S. gordonii Eep. C. albicans microcolonies were cultured using RPMI 1640 medium at 37°C in 5% CO_2_, along with heat-fixed supernatants from S. gordonii (HF s*Sg*) strains (10%) for 17 h, and the biomass was measured by crystal violet straining. Trypsin treatment of HF s*Sg* CH1 significantly reduced *C. albicans* microcolony biomass, while DNase treatment had no effect. HF s*Sg* of CH9278 missing comCDE competence regulatory system had no effect on *C. albicans* microcolony biomass. In contrast, the CH1 Δ*eep* strain missing the zinc metalloprotease Eep significantly reduced *C. albicans* microcolony biomass, and this effect was abolished in the CH1Δ*eep/*pSgEep1 complemented strain. *C. albicans* microcolony biomass was not altered by Todd-Hewitt (TH; 10%) medium alone. Experiments were carried out in duplicates, and the means ± the SD of three independent experiments are shown. Significance was calculated by one-way ANOVA with *post ad hoc* Dunnett’s multiple-comparison test (**, *P* < 0.01).

Many streptococcal signaling molecules known to affect C. albicans biofilm formation are small peptides and competence factors. Therefore, we first treated *S. gordonii* supernatants with trypsin (200 μg/ml) or DNase (500 μg/ml) before heat inactivation. Trypsin treatment significantly reduced microcolony biomass observed by 40%, while DNase treatment had no effect on microcolony formation compared to Todd-Hewitt (TH) medium only (Fig. 6), suggesting that peptides but not competence factors are likely to be responsible for this affect. To further validate these findings, we examined knockout mutants of *S. gordonii* strains defective in the production of secreted heat-stable competence factors (*S. gordonii* CH9278) or pheromone secretion (*S. gordonii* CH1Δ*eep*). Among *S. gordonii* competence factors, the *comCDE* system encodes a sensor-regulator system (ComD ComE), which is activated by the *comC* gene product (CSP, competence stimulating peptide) and regulates the competence response. Deletion of the *comCDE* system has been shown to alter biofilm biomass by increasing extracellular DNA content in C. albicans and S. gordonii mixed biofilms (41). The total fungal biomass of microcolonies grown with HF supernatant of *S. gordonii* CH9278 cells (deficient in comCDE) was not different than microcolonies formed with the parental strain (*S. gordonii* CH1) (Fig. 6). However, the microcolony biomass was significantly decreased by 40% when incubated with HF supernatants of CH1Δ*eep* that are deficient in the zinc metalloprotease Eep required for the processing of pheromones from lipoproteins (42). Furthermore, this effect was specific to Eep, as shown by the restored biomass with the complemented strain CH1Δ*eep/*pSgEep1 (Fig. 6). These results show that an *S. gordonii* heat-resistant, trypsin-sensitive pheromone processed by Eep promotes *C. albicans* hyphal microcolony formation.

### Heat-fixed supernatant of *S. gordonii* increases the expression of *C. albicans* genes required for hyphal morphogenesis while decreasing the expression of adhesion genes.

**FIG 7:**
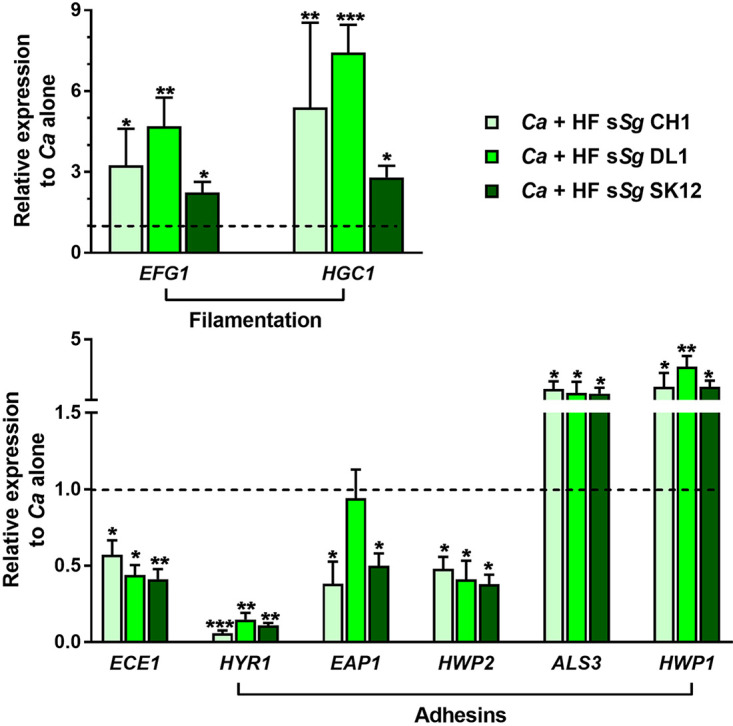
S. gordonii heat-fixed supernatant increases the expression of C. albicans filamentation genes while it decreases the expression of virulence and adhesion genes. C. albicans microcolonies were cultured using RPMI 1640 medium at 37°C in 5% CO_2_, along with heat-fixed supernatant from S. gordonii (HF s*Sg*) strains CH1, DL1, and SK12 for 17 h. Total RNA from microcolonies was isolated, and the relative gene expression obtained by RT-qPCR was normalized to actin and GAPDH. C. albicans microcolonies grown without HF s*Sg* were used as control, and the expression levels of each gene were set as 1. Microcolonies formed in the presence of HF s*Sg* increased the expression of genes required for filamentation (*EFG1* and *HGC1*), as well as cell-cell and cell-substrate adhesion (A*LS3* and *HWP1*), but decreased the expression of other adhesins involved in surface attachment (*HYR1*, *EAP1*, and *HWP2*). Experiments were carried out in duplicate, and graphs indicate the means ± the SD of four independent experiments. Significance was calculated one-way ANOVA with *post ad hoc* Dunnett’s multiple-comparison test (*, *P* < 0.05; **, *P* < 0.01; ***, *P* < 0.001).

Since we observed that incubation with *S. gordonii* HF supernatant increased *C. albicans* microcolony size but decreased yeast adhesion to the substrate, we next measured the expression of genes required for *C. albicans* filamentation (*EFG* and *HGC1*), virulence (*ECE1*), and adhesion (*HYR1*, *EAP1*, *HWP1*, *ALS3*, and *HWP2*) by quantitative reverse transcription-PCR (RT-qPCR). Consistent with increased filamentation and microcolony size induced by *S. gordonii* (Fig. 5), *C. albicans* expression levels of *EFG1* and *HGC1* genes associated with filamentation were significantly increased (2- to 7-fold) in the presence of *S. gordonii* HF supernatant (Fig. 7, upper panel). Intriguingly, we found that the expression of *ECE1*, required for virulence through candidalysin production, was significantly reduced in the presence of *S. gordonii* HF supernatant (Fig. 7, lower panel). Importantly, the expression of *HYR1*, *EAP1*, and *HWP2* adhesins that are involved in initial attachment to surfaces was significantly decreased in the presence of all HF supernatants (Fig. 7, lower panel) and may partially account for the loss of adhesion of microcolonies to the surface that we observed. However, genes involved in some cell-cell and cell-substrate adhesion, including A*LS3* and the hyphal wall protein *HWP1*, were upregulated, consistent with their hypha-specific expression. Together, these data confirm the increase in filamentation induced by *S. gordonii*, as shown by increased hypha-specific gene expression, along with altered expression levels among genes involved in adhesion.

### Heat-fixed supernatants of *S. gordonii* decreased *C. albicans* microcolony adherence to oral epithelial cells while increasing microcolony size.

**FIG 8:**
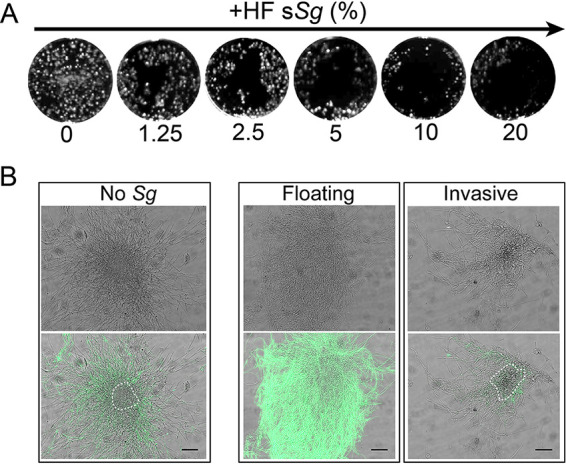
S. gordonii heat-fixed supernatant alters adherence of C. albicans microcolonies to oral epithelial cells. Oral epithelial cells were cultured in glass coverslips with DMEM/F-12 medium supplemented with 10% FBS at 37°C until reaching confluence. C. albicans cells and heat-fixed supernatant from S. gordonii DL1 (HF s*Sg*; 1.25, 2.5, 5, 10, and 20%) were grown together at 37°C in 5% CO_2_ for 17 h to form microcolonies. (A) Microcolony adhesion decreased in the presence of increasing concentrations of HF s*Sg.* (B) Noninvading (floating) C. albicans cells were stained with anti-*C. albicans* primary antibody, followed by Alexa Fluor 488 secondary antibody (shown in green). C. albicans hyphae invading epithelium are indicated by the unstained area (lower panel, white dotted line). The majority of microcolonies formed in the presence of HF s*Sg* had decreased adhesion to epithelial monolayers (floating) compared to microcolonies grown without HF s*Sg*; however, a subset of microcolonies had both high adhesion and invasion. Microcolonies grew on epithelial cells without S. gordonii were used as a control (No *Sg*). Scale bar, 10 μm. Experiments were carried out in duplicate and performed two times. Images represent one biological replicate.

Since adhesion and virulence genes were altered in microcolonies in the presence of *S. gordonii* supernatants, we examined whether the presence of *S. gordonii* would modify *C. albicans* microcolony adherence and invasion with oral epithelial cells. We first grew microcolonies with 1.25 to 20% HF S. gordonii culture supernatants (HF s*Sg*) for 17 h and then gently washed each well to remove nonadherent cells before visualizing adherent microcolonies (Fig. 8A). Even addition of 1.25% of HF s*Sg* resulted in some loss of adhesion compared to microcolonies grown without HF s*Sg* that remained firmly attached and evenly distributed over epithelial monolayers (Fig. 8A). The addition of increasing concentrations of HF s*Sg* resulted in greater detachment of microcolonies until 70% of microcolonies grown with 20% HF s*Sg* were removed by gentle washing. We also observed that among the approximately 30% microcolonies remaining attached with 20% HF s*Sg*, many were much smaller. To determine whether invasion into epithelial monolayers might account for these differences in adhesion, hyphal invasion of microcolonies was assessed (Fig. 8B). Untreated *C. albicans* microcolonies showed a typical phenotype consisting of a large mass of noninvasive hyphae (Fig. 8B, left panel, shown in green) surrounding a central region of hyphae invading epithelium (Fig. 8B, left panel, gray regions indicated by a white dotted line). In contrast, unwashed floating microcolonies grown with 20% HF s*Sg* were much larger and denser than microcolonies grown without HF s*Sg*, and we were unable to detect invasive hyphae within these floating microcolonies. However, among the roughly 30% of small microcolonies grown with HF s*Sg* that remained after washing, nearly 90% of the total area of the microcolony was found to be invading (Fig. 8B, lower right panel, white dotted line). Thus, while the majority of microcolonies grown with HF s*Sg* on epithelium were similar to those formed on a solid substrate exhibiting a large dense floating phenotype, we discovered a subpopulation of microcolonies with a smaller but highly invasive phenotype.

## DISCUSSION

C. albicans and P. aeruginosa are found together in the sputum and lungs of cystic fibrosis patients (14), while S. gordonii and C. albicans are commonly isolated together in various oral biofilms, including prosthetic surfaces in denture stomatitis (10, 43). Macrophages act as immune sentinels performing a variety of functions (44), so that macrophage depletion increased the risk for systemic candidiasis and streptococcal bacterial load, both leading to increased mortality in mice (27, 45). Furthermore, tissue-resident macrophages are important for triggering immune responses in the lung during P. aeruginosa pneumonia (46). Although phagocytic cells are highly effective at controlling infection, pathogenic bacteria and fungi have evolved multiple mechanisms to escape killing. C. albicans hypha formation within macrophages is an escape mechanism to subvert phagosomal clearance (47). Similarly, P. aeruginosa and certain strains of S. gordonii can also resist phagosomal killing in macrophages by promoting autophagy or suppressing phagosomal maturation (34, 48). Our results indicate that C. albicans survival in macrophages in the presence of S. gordonii or P. aeruginosa are not dependent upon changes in macrophage phagocytic uptake, phagosome maturation, or ROS production. Instead, we found that germination and hyphal production induced by coinfecting bacteria were most predictive of fungal survival within macrophages (Fig. 2), so that hyphal expansion results in phagosome membrane damage (49) and eventual escape. Thus, we found that C. albicans escape and subsequent microcolony formation after 17 h was proportional to the degree of hyphal promotion or suppression induced earlier (2 to 3 h) by coinfecting bacteria.

To understand how S. gordonii and P. aeruginosa alter C. albicans filamentation, we examined bacterial effects on microcolony formation outside macrophages and found that the addition of heat-fixed supernatants of S. gordonii significantly altered fungal biomass. Although previous work found that S. gordonii cells promote fungal hyphal morphogenesis via direct contact (50), we found that S. gordonii supernatant alone was effective in promoting microcolony formation. Thus, while physical interaction between fungal and bacterial cells may provide synergy, S. gordonii binding to C. albicans is not required for this effect on filamentation. We also found that S. gordonii competence factors are not involved. Instead, a heat-stable, trypsin-sensitive small peptide processed by the Eep metalloprotease appears to be crucial for hyphal elongation in C. albicans microcolony formation. Although the S. gordonii pheromone autoinducer 2 has been suggested as one diffusible molecule affecting filamentation of C. albicans (7), to our knowledge this is the first instance of an Eep processed pheromone signal peptide affecting fungal growth. In addition to potential lipoprotein signal sequences known to be cleaved by Eep, any protein signal peptide encoded in the S. gordonii genome could potentially be cleaved by Eep, including a large number of unannotated small hydrophobic peptides. Thus, the range of possible targets of Eep processing and the numbers of released peptides are numerous. Eep has similarities to proteases processing certain pheromone precursors in *Enterococcus faecalis* (42, 51); thus, it is likely that Eap may generate related small peptides from other bacteria that may similarly influence C. albicans filamentation. Further studies are needed to determine with more detail the range of bacteria able to influence microcolony formation.

The Dongari-Bagtzoglou group previously showed that the presence of oral streptococci increases the ability of C. albicans to invade organotypic models of oral mucosa (9) and that Streptococcus oralis can activate C. albicans *EFG1* gene expression (a key inducer of hyphae and microcolonies) that increases polymicrobial biofilm (52). In addition, coinfections of S. oralis and C. albicans increase mucosal fungal invasion (53), and coculture of C. albicans with S. gordonii induces the expression of fungal filamentation genes and hyphal adhesins, including Als1, Hyr1, and Eap1 (11), some of which we showed are important for microcolony adhesion (54). However, we found that S. gordonii supernatants induced a dual phenotype, with the majority of C. albicans microcolonies becoming enlarged and detached from an underlying epithelium, while some microcolonies (30%) had less biomass but were highly invasive. This subpopulation of invasive cells may be a result of a microenvironment in which fungal cells have low exposure to secreted bacterial products that cause detachment or, alternatively, some fungal cells might become hyperinvasive in response to S. gordonii when in close contact with epithelium. Further work is needed to understand this phenotype. We found that the floating dense microcolonies had increased in the expression of *ALS3* and *HWP1*, hypha-specific cell adhesion proteins. It is possible that in the context of these S. gordonii modified microcolonies, Als3 and Hwp1 function in cell-to-cell adhesion rather than cell-to-substrate adhesion, resulting in the phenotype of a denser and larger mass of hyphae that are not attached to the underlying surface, potentially increasing dissemination of C. albicans to distant sites. However, S. gordonii might also promote C. albicans commensalism by repressing expression of virulence genes involved in epithelial invasion (*ECE1*) and decreasing expression of genes required for adhesion (*HYR1*, *EAP1*, and *HWP2*). What differences exist in gene expression between these two microcolony phenotypes induced by S. gordonii remains an important question.

In contrast, P. aeruginosa induced a striking reduction of C. albicans microcolony size that was replicated when purified phenazines were added, suggesting that these secreted compounds are responsible for the repression of C. albicans hypha formation and possibly reduced survival within macrophages. Nonlethal concentration of phenazines secreted by P. aeruginosa are known to suppress C. albicans respiration and to acidify the extracellular pH, resulting in suppression of filamentation (40). Important differences have been found among P. aeruginosa clinical isolates in their ability to secrete phenazines and their quorum-sensing inducers (24, 55). The production of phenazines is promoted in environments of low oxygen and nutrient deprivation (56) and by the presence of C. albicans (57), suggesting that these molecules may be more functional in confined spaces, such as the lumens of macrophages while phagocytosed together with C. albicans, compared to the more open environment of respiratory mucosa where phenazines are easily inactivated (58).

Interkingdom signaling is a major contributor in development of the microbiome. The identification of C. albicans genes modified by S. gordonii diffusible factors represents an attractive target to modify virulence of polymicrobial biofilms. Also, potential identification of new fungal pathways affected by S. gordonii or P. aeruginosa may suggest a broad basis for the regulation of fungal biofilms by cocolonizing bacteria.

## MATERIALS AND METHODS

### *C. albicans* and bacterial cultures.

C. albicans and bacteria strains are listed in Table 1. C. albicans CAI-4 or SC5314 wild-type (WT) were cultured overnight in yeast extract-peptone-dextrose (YPD; BD Difco) broth supplemented with 50 μg/ml of uridine (Sigma-Aldrich) at 30°C in an orbital shaker at 220 rpm. Cultures were diluted to an optical density at 600 nm (OD_600_) of 0.3 to 0.4 in fresh YPD medium and allowed to reach an OD_600_ of 0.6 to 0.7. The cells were then washed twice by centrifugation at 2,800 × *g* for 5 min, with phosphate-buffered saline (PBS; pH 7.4; Corning), counted in a hemocytometer, and suspended in the required medium prior to experiments. Heat-killed yeast cells were prepared by incubation at 65°C for 30 min. Strains of S. gordonii were routinely maintained in Todd-Hewitt (TH) agar (BD Biosciences) at 4°C and grown overnight in TH broth at 37°C in 5% CO_2_ for experiments. S. gordonii CH9278 and CH1Δ*eep* strains were maintained in TH agar supplemented with 250 μg/ml spectinomycin (Sigma-Aldrich), and S. gordonii complement CH1Δ*eep/pSgEep1* was kept in TH agar supplemented with 250 μg/ml spectinomycin and 5 μg/ml erythromycin (Sigma-Aldrich). All three mutant strains were grown overnight in TH broth without antibiotics prior to use. P. aeruginosa strains were kept in Luria-Bertani (LB) agar (BD Biosciences) and cultured in broth in an orbital shaker at 220 rpm at 37°C. To evaluate the role of phenazines, P. aeruginosa Δ*phz* strains, characterized by the deletion of the two redundant 7-gene operons *phzA1-phzG1* (*phz1*) and *phzA2-phzG2* (*phz2*) encoding the biosynthetic enzymes responsible for phenazine production, were compared to the PA14 parental strain. Each bacterial culture was grown for 10 h, diluted in fresh medium, and allowed to reach mid-log phase (OD_600_ ≈ 1) prior to use. The cultures were then spun down at 2,500 × *g* for 3 min and washed two times in 1× PBS and counted using a hemocytometer. S. gordonii cells were briefly sonicated for 30 s on ice to break bacterial chains into individual bacteria prior to counting. Fresh-filtered S. gordonii (s*Sg*) or P. aeruginosa (s*Pa*) culture supernatants were collected by centrifugation after log-phase growth and filtered using a 0.20-μm syringe filter (Corning, Inc.). Heat-fixed S. gordonii culture supernatants (HF s*Sg*) were obtained by boiling fresh-filtered supernatant of S. gordonii strains for 15 min and then stored at 4°C until use. When indicated, fresh-filtered S. gordonii supernatant was treated with DNase (500 μg/ml; Sigma-Aldrich) or trypsin (200 μg/ml; Sigma-Aldrich) for 10 min at 37°C with gentle shaking before heat treatment.

**TABLE 1:** Strains used in this study

Organism	Strain	Reference
*Candida albicans*	CAI-4, wild type	61
	SC5314, wild type	62
	CAF2-dTomato	63
*Pseudomonas aeruginosa*	PAO1	64
	94-323-0635 (0635)	64
	PA14	65
	Δ*phz*	65
*Streptococcus gordonii*	CH1	66
	DL1	34
	SK12	67
	CH9278	68
	CH1Δ*eep*	69
	CH1Δ*eep/*pSgEep1	69

### Phagosomal survival and escape of *C. albicans* during coinfection with bacteria.

Survival of C. albicans in macrophage phagosomes was performed as described previously (59) with modifications. Murine RAW 264.7 macrophages from the American Type Culture Collection (ATCC TIB-71) were seeded (5 × 10^5^ cells/ml) in 24-well plates (Corning, Inc.) with RPMI 1640 supplemented with l-glutamine (Corning) and 10% fetal bovine serum (FBS; Seradigm). The cells were activated using 10 ng/ml gamma interferon (BioLegend) for 12 h prior to experiments. C. albicans and bacterial cultures were each grown to mid-log phase as described above, added simultaneously at ratios of 10 macrophages:1 C. albicans:1 bacteria (MOI of 0.1:0.1) or 10 macrophages:1 C. albicans:10 bacteria (MOI of 0.1:1), and then incubated for 3 h at 37°C and 5% CO_2_. For survival assays, 0.25% SDS (Thermo Fisher Scientific) and sterile water were added to lyse macrophages and release phagocytosed C. albicans and bacteria. Lysates were serially diluted and cultured for 24 h at 30°C on yeast-dextrose-peptone agar supplemented with 50 μg/ml streptomycin and 50 U/ml penicillin (Sigma-Aldrich) to remove bacteria and obtain C. albicans CFU. The percentage of survival was determined as follows: (recovered C. albicans CFU after phagocytic cell lysis/total number of phagocytosed C. albicans) × 100. For escape assays, 10 macrophages:1 C. albicans:1 bacteria (MOI of 0.1:0.1) were incubated together for 3 h at 37°C and 5% CO_2_ to allow for phagocytosis of yeast and bacterial cells, and then 50 μg/ml streptomycin and 50 U/ml penicillin (Sigma-Aldrich) were added to each well to suppress the growth of nonphagocytosed bacteria or bacteria released by the lysis of macrophages. Macrophage monolayers were incubated at 37°C in 5% CO_2_ for 17 h to permit visualization of C. albicans able to form microcolonies. Microcolonies on the surface of macrophage monolayers were imaged using a Zeiss Axio microscope, and the total number of microcolonies per well was counted. Assays were performed in duplicates, and results are representative of at least three independent experiments.

### Evaluation of phagosome maturation in mouse macrophages.

Macrophages were seeded in 12-well culture plates with cover glass (Azer Scientific) and activated as described above. Phagosomal maturation was measured (34) with some modifications. C. albicans and bacterial cells were counted, and added to macrophages at an MOIs of 1 and 10, respectively. Nonphagocytosed C. albicans cells were stained with calcofluor white (CW; Sigma-Aldrich) for 2 min on ice to differentiate them from phagocytosed cells. Cover glasses were washed, returned to fresh RPMI 1640 medium, and maintained at 37°C. Cells were sampled at indicated time points and immunostained for lysosomal associated membrane protein 1 (LAMP1) (clone 1D4B; Developmental Studies Hybridoma Bank, University of Iowa) or early endosomal antigen 1 (EEA1; Cell Signaling Technology) and Alexa 594-conjugated donkey anti-rat or anti-rabbit secondary antibody (Jackson Immunoresearch). For dextran labeling, macrophages were incubated with Alexa 594-dextran (Invitrogen) with a chase of 2 h to ensure all dextran was in lysosomes prior to performing phagocytosis experiments as described above. In all cases, macrophages were costained with Alexa Fluor 488-conjugated phalloidin (Invitrogen). At least 50 phagosomes were observed per time point using a Zeiss Axio Observer Z1 inverted fluorescence microscope (Carl Zeiss, Germany) and ZEN 2011 (blue edition) software. Phagosomes of internalized C. albicans (no CW stain) with total surrounding staining were counted as positive, and phagosomes with incomplete or nonexistent staining were counted as negative. The percentage of label-positive phagosomes was calculated as the number of positive phagosomes/total number of phagosomes counted × 100.

### Evaluation of hypha formation within macrophages.

Fluorescent C. albicans CAF2-dTomato and unlabeled bacterial strains were added to macrophages at MOIs of 0.5 and 5, respectively, spun to allow contact, and incubated at 37°C and 5% CO_2_ for 2 h. These MOIs were used so that some yeast and bacterial cells would not be phagocytosed and the formation of hyphae extracellularly could be compared to that of phagocytosed yeast. The phagocytic index for C. albicans cells was evaluated as previously described (59) with or without added bacteria to ensure that bacteria did not alter the phagocytic indices of yeast cells. Extracellular C. albicans was stained with CW, cells were fixed with 4% paraformaldehyde, and then macrophages were permeabilized and stained with phalloidin. A minimum of 100 phagocytosed *Candida* cells was counted using a Zeiss Axio microscope and classified as yeast or hyphae. The percentage of hyphal cells was calculated by obtaining the ratio of total hyphal form C. albicans/total number of *Candida* counted × 100.

### *C. albicans* microcolony formation.

C. albicans microcolonies were formed by seeding 100 cells in 12-well plates using RPMI 1640. Whole *S. gordonii* or *P. aeruginosa* bacterial strains (1,000 cells per well), s*Sg* or s*Pa* (10% final volume), or heat-fixed (HF) s*Sg* (1.25 to 20% final volume) were added to wells, followed by incubation at 37°C and 5% CO_2_ for 17 h to form microcolonies. For experiments with phenazines, purified pyocyanin (30 mM) (PYO; Cayman Chemical) and phenazine methosulfate (25 mM; PMS; Acros Organic) were diluted in H_2_O to reach a final concentration after addition to wells (PYO [20 μM] and PMS [5 μM]). Microcolonies were imaged, and density was measured with ImageJ using inverse gray values/μm^2^ (2). The biomass of microcolonies grown with HF s*Sg* was determined by crystal violet (CV) staining as described previously (60). The total absorbance (*A*_595_) was obtained by subtracting negative controls (no cells) from experimental samples of destained solutions using a FlexStation 3 multimode microplate reader (Molecular Devices).

### Microcolony invasion on oral epithelial cells.

TR146 epithelial cells, a buccal epithelial squamous carcinoma cell line from the European Collection of Authenticated Cell Cultures (ECACC), were grown on glass coverslips to confluence in 1:1 Dulbecco modified Eagle medium (DMEM)/F-12 medium supplemented with 10% FBS. Invasion of epithelial cells by C. albicans microcolonies was performed as described previously (2) with or without 10% HF s*Sg*. After 17 h of incubation, microcolonies were photographed with white light using an InGenius imaging system (Syngene). The medium covering the cells was then aspirated, and monolayers were washed and stained as described previously (2).

### Microcolony RNA isolation and qRT-PCR.

C. albicans microcolonies formed in the presence of HF s*Sg* were collected and pelleted by centrifugation at 10,000 × *g* for 5 min to isolate RNA as previously described (2). Total RNA was further purified by using an RNeasy minikit (Qiagen, Hilden, Germany) and quantified with a NanoDrop One (Thermo Scientific). Total RNA was used to quantitate *HWP1*, *HWP2*, *ECE1*, *HYR1*, *EAP1*, *ALS3*, *EFG1*, and *HGC1* gene expression using primers listed in Supplemental Table S1. Total cDNA was synthesized using iScript cDNA synthesis kit (Bio-Rad). All samples were prepared with SsoAdvanced Universal SYBR Green Supermix (Bio-Rad) and cycled using a CFX Connect Real time system (Bio-Rad). Data were analyzed with CFX Maestro software (Bio-Rad). The relative fold changes in gene expression were calculated using both C. albicans actin and GAPDH (glyceraldehyde-3-phosphate dehydrogenase) as controls.

10.1128/mSphere.00689-20.3 TABLE S1 Primers used in this study. Download Table S1, DOCX file, 0.02 MB. Copyright © 2020 Salvatori et al. 2020 Salvatori et al. This content is distributed under the terms of the Creative Commons Attribution 4.0 International license.

### Statistical analysis.

Data were analyzed by a Student *t* test or one-way analysis of variance (ANOVA) with a *post ad hoc* Dunnett’s multiple-comparison test using Prism v7 (GraphPad Software, La Jolla, CA) at a significance level of *P* < 0.05 for all experiments.
