# Supplementary material for: Bacteria Modify Candida albicans Hypha Formation, Microcolony Properties, and Survival within Macrophages
Source: mSphere. 2020 Aug 5;5(4):e00689-20. doi: 10.1128/mSphere.00689-20 (PMC7407070; doi:10.1128/mSphere.00689-20)
Supplement: TABLE S1 [file mSphere.00689-20-st001.docx]

**TABLE S1.** Primers used in this study

| ***HWP1*** | *HWP1*qRTForward: CTCCTGCCACTGAACCTTCC  *HWP1*qRTReverse: GAGCCAGCTGGAGCAGTTT |
| --- | --- |
| ***HWP2*** | *HWP2*qRTForward: CTGTCCTCTTACCGATACA  *HWP2*qRTReverse: GAACTAGTGACAGACGTAGA |
| ***ECE1*** | *ECE1*qRTForward: TTGCTAATGCCGTCGTCAGA  *ECE1*qRTReverse: CCAGGACGCCATCAAAAACG |
| ***HYR1*** | *HYR1*qRTForward: GCTGCTGCCCTTCCACAATA  *HYR1*qRTReverse: GGTGCAGATGGTCCATTGGT |
| ***EAP1*** | *EAP*1qRTForward: CAAGGTCAAGCCCATTAC  *EAP*1qRTReverse: CGTGTAGGAGGTAGATTCA |
| ***ALS3*** | *ALS3*qRTForward: GAGACCGATACTGTCCTTAT  *ALS3*qRTReverse: GGAGGAGCAGTGATTGTA |
| ***EFG1*** | *EFG1*qRTForward: CCCCCATACTTCCAATTCT  *EFG1*qRTReverse: CTCGTGGTCTGATTCCTGGT |
| ***HGC1*** | *HGC1*qRTForward: CACCACCACAAATGCATTCTCA  *HGC1*qRTReverse: ATGAGGTGCAGGAAGCTGAC |
| ***ACT1*** | *ACT1*qRTForward: ACTGCTTTGGCTCCATCTTCT  *ACT1*qRTReverse: TGGATGGACCAGATTCGTCG |
| ***GAPDH*** | *GAPDH*qRTForward: CGGTCCATCCCACAAGGA  *GAPDH*qRTReverse: AGTGGAAGATGGGATAATG |

All samples were prepared with 1 μl of cDNA template in 20 μl reactions and 150 nM of forward and reverse primers. Samples were denatured for 3 min at 95°C, then, reactions were cycled 40 times using the following parameters: 95°C for 30 s, 50°C for 30 s, and 72°C for 30 s.
